# Secondary healthcare costs after mastectomy and immediate breast reconstruction for women with breast cancer in England: population-based cohort study

**DOI:** 10.1093/bjs/znad149

**Published:** 2023-06-12

**Authors:** Syed Mohiuddin, William Hollingworth, Joel Glynn, Tim Jones, Leigh Johnson, Shelley Potter, Brighter Study Group, Chris Holcombe, Joe O’Donoghue, John Browne, Carmel Gulliver-Clarke, Ranjeet Jeevan, Paul White, Mairead Mackenzie, Patricia Fairbrother

**Affiliations:** Population Health Sciences, Bristol Medical School, University of Bristol, Bristol, UK; Population Health Sciences, Bristol Medical School, University of Bristol, Bristol, UK; NIHR ARC West, University Hospitals Bristol and Weston NHS Foundation Trust, Bristol, UK; Population Health Sciences, Bristol Medical School, University of Bristol, Bristol, UK; Population Health Sciences, Bristol Medical School, University of Bristol, Bristol, UK; NIHR ARC West, University Hospitals Bristol and Weston NHS Foundation Trust, Bristol, UK; Population Health Sciences, Bristol Medical School, University of Bristol, Bristol, UK; Translational Health Sciences, Bristol Medical School, University of Bristol, Bristol, UK; Bristol Breast Care Centre, Southmead Hospital, Bristol, UK

## Abstract

**Background:**

Immediate breast reconstruction after mastectomy can improve the quality of life for women with breast cancer and rates are increasing. Long-term inpatient costs of care were estimated to understand the impact of different immediate breast reconstruction procedures on healthcare expenditure.

**Methods:**

Hospital Episode Statistics Admitted Patient Care data were used to identify women undergoing unilateral mastectomy and immediate breast reconstruction in English National Health Service hospitals (1 April 2009 to 31 March 2015) and any subsequent procedures performed to revise, replace, or complete the breast reconstruction. Costs were assigned to Hospital Episode Statistics Admitted Patient Care data using the Healthcare Resource Group 2020/21 National Costs Grouper. Generalized linear models were used to estimate mean cumulative costs for five immediate breast reconstruction procedures over 3 and 8 years, adjusting for covariates (age/ethnicity/deprivation).

**Results:**

A total of 16 890 women underwent mastectomy and immediate breast reconstruction: implant (5192; 30.7 per cent), expander (2826; 16.7 per cent), autologous latissimus dorsi flap (2372; 14.0 per cent), latissimus dorsi flap with expander/implant (3109; 18.4 per cent), and abdominal free-flap reconstruction (3391; 20.1 per cent). The mean (95 per cent c.i.) cumulative cost was lowest for latissimus dorsi flap with expander/implant reconstruction (€20 103 (€19 582 to €20 625)) over 3 years and highest for abdominal free-flap reconstruction (€27 560 (€27 037 to €28 083)). Over 8 years, expander (€29 140 (€27 659 to €30 621)) and latissimus dorsi flap with expander/implant (€29 312 (€27 622 to €31 003)) reconstructions were the least expensive, while abdominal free-flap reconstruction (€34 536 (€32 958 to €36 113)) remained the most expensive, despite having lower costs for revisions and secondary reconstructions. This was driven primarily by the cost of the index procedure (€5435 (expander reconstruction) to €15 106 (abdominal free-flap reconstruction)).

**Conclusion:**

Hospital Episode Statistics Admitted Patient Care Healthcare Resource Group data provided a comprehensive longitudinal cost assessment of secondary care. Although abdominal free-flap reconstruction was the most expensive option, higher costs of the index procedure need to be balanced against ongoing long-term costs of revisions/secondary reconstructions, which are higher after implant-based procedures.

## Teaser

Immediate breast reconstruction is performed to improve outcomes after mastectomy for breast cancer, but the long-term costs of different breast reconstruction procedures are also an important consideration. The costs of the five most commonly performed breast reconstruction procedures were compared at 3 and 8 years after the index procedure in a large population-based cohort. Abdominal free-flap reconstruction was the most expensive reconstruction option overall at 8 years due to the high cost of the index procedure, but the costs of additional surgery were low, while prosthetic reconstructions were less expensive, but associated with much higher costs for revisions and secondary reconstructions, which may make them the most expensive options longer term.

## Introduction

Breast cancer is the most common type of cancer in women, with an estimated 2.3 million new cases and 685 000 deaths globally in 2020^1^. In the UK, there are around 55 200 new cases of breast cancer each year^2^. Around one in seven UK women will develop breast cancer in their lifetime, and the incidence is projected to rise by 2 per cent in the UK between 2014 and 2035^2^. Breast cancer survival is increasing; almost 80 per cent of the 55 000 women diagnosed with breast cancer each year in the UK survive at least 10 years after their diagnosis and 67 per cent survive 20 years or more^2^. Despite advances in breast cancer treatment and care, up to 40 per cent of women in the UK require a mastectomy^3^. The loss of a breast can adversely affect women’s quality of life and impact personal, sexual, and social relationships^4,5^.

In the UK, breast reconstruction is offered to improve the quality of life after mastectomy and rates of immediate breast reconstruction (IBR) are increasing^6^. Reconstruction options can broadly be categorized as: expander/implant-based reconstruction; or autologous reconstruction that can be performed either with pedicled flaps (for example latissimus dorsi (LD) flaps) or more complex free flaps^7^. Breast reconstruction procedures have different short-term risks and benefits, such as duration of surgery, length of hospital stay and recovery after surgery, surgical complications, and number and position of scars^7^. Expander/implant-based breast reconstruction offers relatively rapid recovery, with no additional scarring, but patients’ satisfaction with the outcome may decrease over time^8^. A significant proportion of women may require further surgery that may have substantial resource implications for the health system and be an additional burden for patients. Women electing to undergo initially more complex and resource-intensive autologous breast reconstruction may require less additional surgery^9^ over time and may be more satisfied with the long-term outcomes of their surgery than those who undergo expander/implant -based breast reconstruction^8,10^.

Policymakers and women require high-quality evidence comparing the long-term costs and outcomes of implant-based and autologous breast reconstruction options to inform shared decision-making^11–13^. This has recently been identified as a key research priority^14,15^. The few studies that have compared the costs of IBR options have produced mixed results^16,17^. Most studies are based on experience at single centres with a relatively short follow-up. RCTs are needed to determine the long-term clinical and cost-effectiveness of different approaches to breast reconstruction^14^, but RCTs comparing types of reconstruction methods have not been feasible due to patient and surgeon preference^18,19^. Routinely collected data for population health research may offer a timely and efficient approach for estimating the long-term costs of breast reconstruction.

This work is part of the wider Brighter study^20^ investigating the long-term clinical and cost-effectiveness of breast reconstruction. The aim of this paper was to use routine healthcare cost data from a large nationally representative population-based cohort to compare the long-term inpatient costs of care of five commonly performed IBR procedures and to describe the costs attributable to the initial surgical reconstruction procedure, as well as revision, secondary reconstruction, completion surgery, adjuvant therapy, and other inpatient admissions at 3 and 8 years after the initial procedure.

## Methods

### Data sources

Hospital Episode Statistics Admitted Patient Care (HES-APC) contains data on all inpatient and day-case admissions funded by the National Health Service (NHS) in England^21^. Data, extracted from medical records by clinical coders, include patient characteristics, diagnosis and procedure codes, and admission and discharge dates. HES-APC data are collated centrally by NHS Digital and are frequently used for research due to its comprehensive coverage of longitudinal data for large numbers of patients. Anonymized patient-level HES-APC data were obtained for all women aged 16 years or over, who had undergone a unilateral mastectomy for invasive breast cancer or ductal carcinoma *in situ* (DCIS) in an NHS England setting between 1 April 2009 and 31 March 2015. All diagnosis and procedure codes used to identify the cohort are summarized in *Table S1*.

### Study population

Women were considered to have undergone IBR if the mastectomy code was accompanied by a code for a reconstructive procedure, performed on the same side and on the same day as the index mastectomy. IBR procedures were classified into five groups: implant only; tissue expander (expander); autologous pedicled LD flap without expander/expander (ALD flap); pedicled LD flap with expander/implant (LD flap + implant); and abdominal free flap (AFF). Women who had less commonly performed breast reconstruction procedures, including transverse rectus abdominis myocutaneous (TRAM) flaps and gluteal flaps, were excluded. These groups were uncommon (less than 1.2 per cent), thus precluding meaningful analysis.

Implant and expander breast reconstruction groups were considered separately because the practice of prosthetic reconstruction changed over the study interval. Acellular dermal matrices and other mesh products were introduced, facilitating single-stage direct-to-implant reconstruction by allowing a definitive fixed-volume implant to be placed at the first operation. It was hypothesized that long-term surgical costs after insertion of a single-stage implant as a definitive reconstruction may differ from two-stage procedures requiring insertion of an expander then an implant.

### Revisions, secondary reconstructions, and completion procedures

Revisions were defined as any ipsilateral procedure performed to the index breast reconstruction to improve the appearance of the reconstruction and/or correct complications after the patient had been discharged after their index procedure. A list of procedure codes was developed and refined iteratively in collaboration with expert breast and plastic surgeons and the existing literature (*Table S2*). Procedures performed during the initial inpatient stay were considered to address immediate postoperative complications. These increased the length of stay and cost of the index admission, but were not categorized as revision procedures in the analysis.

Secondary reconstructions were defined as the replacement of the index breast reconstruction with another, usually different, type of reconstruction with or without the removal of the index reconstruction. Women who underwent a subsequent implant/expander-based reconstruction having had an interval without a reconstruction (reconstruction failure) were considered to have undergone a secondary reconstruction (*Table S3*). Women who underwent an exchange of implant/expander, in which one implant/expander was removed, but immediately replaced with another prosthesis, were considered to have had a revision of their reconstruction rather than a secondary reconstruction. Symmetrization to the contralateral breast (reduction, mastopexy, or augmentation) and nipple/areolar reconstruction were categorized as procedures for ‘completion’ of the reconstructive process (*Table S4*).

Three additional categories were created to group episodes that did not fall into any of the above. The first category captured ‘other breast procedures’ that were not revisions, secondary reconstructions, or completions (episodes with the Healthcare Resource Group (HRG) code starting ‘JA’ that had not already been categorized). The second category captured ‘adjuvant therapies’ (for example chemotherapy or radiotherapy) with the initial HRG codes of ‘SC’ or ‘SB’. The final category, ‘other hospital admissions’, captured all remaining episodes (potentially unrelated to breast cancer).

### Length of follow-up

Complete HES-APC data were available up to 31 March 2019, such that women had between 4 and 10 years of follow-up after initial breast reconstruction. HES-APC only captures the date of death if death occurs in hospital. For these women, survival years after breast reconstruction were calculated.

### Measurement of costs

HES-APC records all finished consultant episodes (FCEs), with each representing an interval of care in hospital under one consultant. Therefore, a spell in hospital may comprise more than one consecutive FCE. The initial breast reconstruction admission and length of stay was defined as the index admission, plus, if applicable, hospital transfers for further care. Length of stay was calculated as the difference between the date of admission for the index mastectomy with IBR and the final date of discharge from an NHS hospital. Based on HES-APC data, HRG codes were assigned to each FCE using ‘HRG4+ 2020/21 National Costs Grouper’ software^22^. HRG codes group clinically comparable treatments that use a broadly similar amount of NHS resources. Each FCE was assigned a cost (in British Pounds) based on these HRG codes using the ‘National Schedule of NHS Costs 2018/19’^23^, and converted to Euros (using the exchange rate £1 = €1.1967 on 17 January 2022). These costs include the costs of surgery, breast implants, and care while on the inpatient wards. Notably, however, since 2012, acellular dermal matrix costs have been excluded from national NHS reference costs; instead hospitals locally negotiate separate reimbursement. Therefore, these costs are not included in the analyses.

### Statistical methods

For each breast reconstruction procedure, the mean cumulative cost of inpatient care per woman was estimated over intervals of 3 and 8 years of follow-up after the initial operation, to explore whether the initial cost and burden of more complex breast reconstruction procedures is offset by reduced need for subsequent surgery. Costs were also stratified by admission type (initial procedure, revision, secondary reconstruction, completion, other breast procedure, adjuvant therapy, and any other hospitalization) over 3 and 8 years, to describe the contribution of each admission type to the total cost.

Furthermore, regression models were used to estimate the mean cumulative cost of inpatient care (including the index admission) for each IBR procedure over 3 and 8 years. Specifically, the inpatient healthcare costs were regressed on each breast reconstruction option, adjusting for age, ethnicity (white/other), socio-economic deprivation (that is index of multiple deprivation quartile^24^), disease status (breast cancer/DCIS), Charlson co-morbidity score^25^, and time until death (if applicable) since the initial reconstruction. Generalized linear models (GLMs) were used to estimate the costs and 95 per cent confidence intervals. The rationale for using GLM regression for healthcare cost data is described elsewhere^26–28^. A Box-Cox test and a modified Park test were used to identify the most appropriate link and distribution function for the GLM^28^. These tests supported the use of the log link and the gamma distribution. Robust standard errors were used in all models to allow for potential misspecification. Analyses were conducted using Stata version 16.1.

## Results

**Fig. 1:**
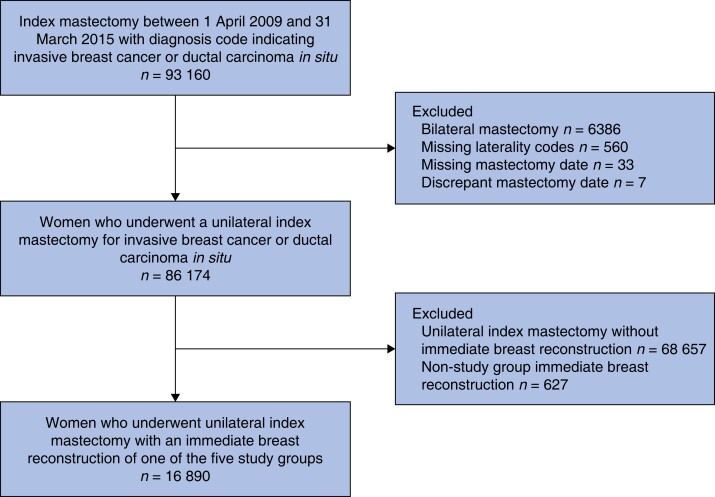
Flow chart of patient records included in the analysis

Mastectomy records with diagnostic codes indicating invasive breast cancer or DCIS were identified for 93 160 women at hospitals in England between 1 April 2009 and 31 March 2015 (Fig. 1). Of these, records were excluded if they did not contain laterality codes (560; 0.6 per cent) or indicated bilateral mastectomy (6386; 6.9 per cent). A further 40 records were excluded due to a missing (33/40) or discrepant (7/40) mastectomy date. Of the 86 174 women undergoing a unilateral mastectomy for invasive or preinvasive breast cancer, 16 890 (19.6 per cent) underwent an IBR with implant-only (5192; 30.7 per cent), expander-based (2826; 16.7 per cent), ALD flap (2372; 14.0 per cent), LD flap + implant (3109; 18.4 per cent), or AFF (3391; 20.1 per cent) reconstructions.

The demographics of the 16 890 patients included in the study cohort are summarized in Table 1. The mean (s.d.) age was 52.6 (9.9) years at the time of mastectomy; 15 020 (88.9 per cent) were of white ethnicity. Some 13 294 (78.7 per cent) had invasive breast cancer and 3596 (21.3 per cent) had preinvasive disease (DCIS). The reconstructions performed changed over time; implant-only and AFF reconstructions became more popular in later years and LD flaps with or without implants were performed less frequently. A slightly lower proportion of women who had AFF reconstruction (596/3391; 17.6 per cent) resided in the most deprived areas compared with women who had expander-based reconstruction (648/2826; 22.9 per cent) (Table 1).

**Table 1:** Patient characteristics at the time of surgery stratified by breast reconstruction type

	Implant (*n* = 5192)	Expander (*n* = 2826)	ALD flap (*n* = 2372)	LD flap + implant (*n* = 3109)	AFF (*n* = 3391)	All (*n* = 16 890)	*P*
Age at index mastectomy (years), mean (s.d.)	52.86(10.57)	51.86(10.30)	53.28(9.87)	52.53(9.74)	52.15(8.54)	52.55(9.90)	<0.001
**Year of mastectomy**							<0.001
2009–2011	1578 (30.39)	1229 (43.49)	1298 (54.72)	1732 (55.71)	1367 (40.31)	7204 (42.65)	
2012–2015	3614 (69.61)	1597 (56.51)	1074 (45.28)	1377 (44.29)	2024 (59.69)	9686 (57.35)	
**Ethnicity**							<0.001
White	4558 (87.79)	2576 (91.15)	2153 (90.77)	2869 (92.28)	2864 (84.46)	15 020 (88.93)	
Other	426 (8.20)	147 (5.20)	155 (6.53)	160 (5.15)	425 (12.53)	1313 (7.77)	
Not known	208 (4.01)	103 (3.64)	64 (2.70)	80 (2.57)	102 (3.01)	557 (3.30)	
Deprivation quintile of residence*							<0.001
1 (most deprived)	1036 (19.95)	648 (22.94)	499 (21.04)	600 (19.30)	596 (17.59)	3379 (20.01)	
2	1077 (20.74)	554 (19.61)	446 (18.80)	593 (19.07)	706 (20.84)	3376 (19.99)	
3	1052 (20.26)	499 (17.66)	483 (20.36)	632 (20.33)	711 (20.99)	3377 (20.00)	
4	977 (18.82)	572 (20.25)	481 (20.28)	644 (20.71)	703 (20.75)	3377 (20.00)	
5 (least deprived)	1050 (20.22)	552 (19.54)	463 (19.52)	640 (20.59)	672 (19.83)	3377 (20.00)	
**Disease status**							<0.001
Invasive cancer	4115 (79.26)	2320 (82.09)	1915 (80.73)	2401 (77.23)	2543 (74.99)	13 294 (78.71)	
DCIS	1077 (20.74)	506 (17.91)	457 (19.27)	708 (22.77)	848 (25.01)	3596 (21.29)	
Charlson co-morbidity score, mean (s.d)	0.23(0.50)	0.22(0.53)	0.21(0.50)	0.19(0.48)	0.19(0.45)	0.21(0.49)	<0.001
Follow-up interval (days), mean(s.d.)	2321.75(610.04)	2535.91(612.34)	2689.92(613.99)	2687.49(590.15)	2466.34(631.26)	2505.64(629.14)	<0.001

Values are *n* (%) unless otherwise indicated. *Based on ‘2019 Index of Multiple Deprivation’ rank of lower super output area of residence. ALD flap, autologous latissimus dorsi flap; LD flap + implant, latissimus dorsi flap with implant/expander; AFF, abdominal free flap; DCIS, ductal carcinoma *in situ*.

Table 2 presents the mean (95 per cent c.i.) total cost of care over 3 and 8 years by each breast reconstruction procedure type. Adjusting for covariates, the mean total cost of care per woman was lowest for LD flap + implant reconstruction (€20 103 (95 per cent c.i. €19 582 to €20 625)) over 3 years, while highest for AFF reconstruction (€27 560 (95 per cent c.i. €27 037 to €28 083)). Over 8 years, the mean total cost of care per woman was lowest for expander-based reconstruction (€29 140 (95 per cent c.i. €27 659 to €30 621)), while highest for AFF reconstruction (€34 536 (95 per cent c.i. €32 958 to €36 113)). The mean total cost from 3 to 8 years, however, increased by the least amount for AFF reconstruction (€6976) and by the most amount for implant-only reconstruction (€9521). The number of observations at 8 years of follow-up ranged between 842 (expander reconstruction) and 1235 (LD flap + implant reconstruction).

**Table 2:** Total cost of inpatient care per woman over 3 and 8 years by breast reconstruction type

Immediate breast reconstruction type	Length of follow-up (years)	Number of observations	Cost (€), mean (95% c.i.)*
Implant	3	5192	20 778 (20 331,21 226)
	8	1074	30 299 (28 649,31 948)
Expander	3	2826	21 667 (21 124,22 211)
	8	842	29 140 (27 659,30 621)
ALD flap	3	2372	20 984 (20 370,21 598)
	8	993	29 889 (28 149,31 630)
LD flap + implant	3	3109	20 103 (19 582,20 625)
	8	1235	29 312 (27 622,31 003)
AFF	3	3391	27 560 (27 037,28 083)
	8	950	34 536 (32 958,36 113)

*These costs are the means of predicted costs from the generalized linear model regression if every woman in the cohort had each type of index operation (implant, expander, etc.). ALD flap, autologous latissimus dorsi flap; LD flap + implant, latissimus dorsi flap with implant/expander; AFF, abdominal free flap.

Table 3 presents the mean (95 per cent c.i.) stratified cost per woman over 3 and 8 years by admissions for different reasons. The cost of the initial breast reconstruction procedure was higher for women undergoing autologous reconstructions. Specifically, AFF reconstruction had a higher initial cost (€15 106 (95 per cent c.i. €15 060 to €15 153)) than expander-based (€5435 (95 per cent c.i. €5377 to €5493)) or implant-only (€6457 (95 per cent c.i. €6411 to €6505)) reconstructions. Over the follow-up interval of 8 years, the costs for revisions and secondary reconstructions, however, tended to be lowest for women initially receiving autologous reconstructions. For example, the total cost of revision procedures after AFF reconstruction (€2433 (95 per cent c.i. €2146 to €2721)) was less than half that after expander-based reconstruction (€5642 (95 per cent c.i. €5317 to €5968)). This reflects the fact that by 8 years, 86 per cent of patients in the expander group and 70 per cent of those in the implant-only group had undergone at least one revision procedure compared with only 47 per cent of those in the AFF group (data not shown). As for the total cost of secondary reconstruction procedures, AFF reconstruction (€351 (95 per cent c.i. €224 to €479)) was significantly less costly than the expander-based (€2308 (95 per cent c.i. €1990 to €2626)) and implant-only (€2000 (95 per cent c.i. €1722 to €2276)) reconstructions. The total cost of completion procedures, however, was highest for AFF reconstruction (€1601 (95 per cent c.i. €1453 to €1750)) and lowest for implant-only reconstruction (€939 (95 per cent c.i. €823 to €1057)).

**Table 3:** Cost (€) per woman over 3 and 8 years by admission for each breast reconstruction type

	Implant	Expander	ALD flap	LD flap + implant	AFF
Index procedure	6457 (6411,6505)	5435 (5377,5493)	7785 (7753,7816)	7840 (7811,7868)	15 106 (15 060,15 153)
**Revision procedure**					
3 years	2861 (2757,2967)	4380 (4238,4524)	2220 (2074,2366)	2216 (2099,2332)	1885 (1772,1997)
8 years	4163 (3868,4459)	5642 (5317,5968)	2864 (2568,3159)	3266 (3017,3516)	2433 (2146,2721)
**Secondary reconstruction procedure**					
3 years	1075 (982,1168)	1793 (1636,1949)	201 (148,255)	293 (233,353)	278 (217,339)
8 years	2000 (1722,2276)	2308 (1990,2626)	456 (314,597)	527 (387,667)	351 (224,479)
**Completion procedure**					
3 years	668 (625,712)	725 (668,781)	898 (832,965)	972 (912,1032)	1264 (1199,1327)
8 years	939 (823,1057)	1193 (1040,1346)	1148 (1029,1266)	1254 (1142,1368)	1601 (1453,1750)
**Other breast-related procedure**					
3 years	1371 (1286,1456)	1318 (1203,1432)	1058 (953,1162)	1063 (971,1155)	833 (755,909)
8 years	2210 (1948,2472)	1972 (1680,2265)	1768 (1539,1996)	1728 (1531,1925)	1422 (1228,1616)
**Adjuvant therapy**					
3 years	5112 (4763,5463)	4813 (4399,5227)	5067 (4610,5525)	4614 (4203,5021)	5145 (4723,5565)
8 years	6821 (5623,8019)	5385 (4374,6396)	7295 (6089,8501)	7502 (6124,8881)	6869 (5657,8081)
**Other hospitalization**					
3 years	3237 (3058,3418)	3205 (2980,3429)	3759 (3475,4044)	3113 (2879,3346)	3067 (2861,3273)
8 years	8122 (7280,8964)	7487 (6767,8207)	8543 (7635,9450)	7181 (6352,7831)	6797 (6157,7439)

Values are mean (95% c.i.). ALD flap, autologous latissimus dorsi flap; LD flap + implant, latissimus dorsi flap with implant/expander; AFF, abdominal free flap.

**Fig. 2:**
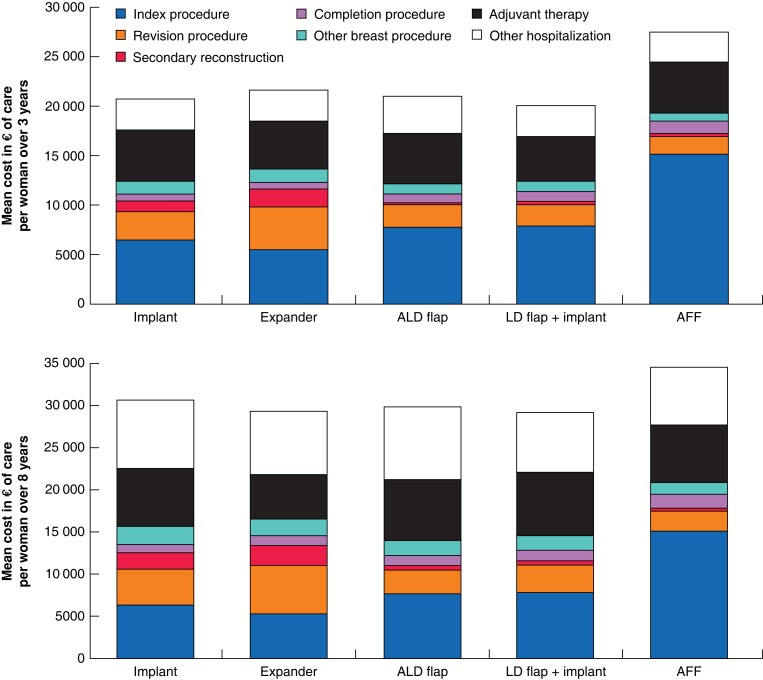
Mean cost of care per woman over 3 and 8 years by admission for each breast reconstruction type ALD flap, autologous latissimus dorsi flap; LD flap + implant, latissimus dorsi flap with implant/expander; AFF, abdominal free flap.

AFF reconstruction was associated with the highest overall healthcare costs over both 3 and 8 years, driven primarily by the high cost associated with the index breast reconstruction procedure (Fig. 2).

## Discussion

IBR after mastectomy is offered to improve the quality of life for women with breast cancer and the rates of IBR are increasing. Different reconstruction procedures vary with regard to the rates of short- and long-term complications, the need for revisions and secondary reconstructions, and patient satisfaction with the outcomes of surgery. This study used nationally representative population-based cohort data of 16 890 women to provide novel insights into the comparative long-term impact of different breast reconstruction procedures on the costs to the NHS.

Over 3 years after initial surgery, the lowest overall cost of care was seen in women undergoing LD flap + implant (€20 103) and implant-only (€20 778) reconstructions, with AFF reconstruction (€27 560) representing the most expensive reconstruction option. By 8 years, the overall costs of prosthetic reconstructions and LD flaps with and without implants were broadly similar (expander €29 140, implant €30 299, ALD flap €29 889, and LD flap + implant €29 312). AFF reconstruction (€34 536) remained the most expensive reconstruction option due to the high cost of the index procedure, even though the costs of revisions and secondary reconstructions were much lower than for other breast reconstruction procedure types. The marked difference in the costs of revisions and secondary reconstructions between AFF and expander/implant-based reconstructions, however, is a key finding, as the numbers of revisions and secondary reconstructions are likely to continue to increase over time in the prosthetic reconstruction group, further adding to the procedure costs. This may mean that longer-term, implant-based procedures become the most expensive reconstruction option, but further long-term data are needed. Such future work is vital, as implant-only and AFF reconstructions are now the main types of IBR performed in the UK and both the short- and long-term costs associated with this change in practice will require careful consideration along with patient satisfaction to inform decision-making.

Several studies have attempted to compare the healthcare costs of different breast reconstruction procedures, but generated conflicting results^17,29,30^. In keeping with the results of the current study, Aliu *et al*.^29^ reported a higher 2-year cumulative cost of care for autologous compared with implant-based reconstruction. Fischer *et al*.^30^ reported a higher 3-year cumulative cost of care for autologous reconstruction compared with implant-only and expander-based reconstructions. The authors, however, highlighted the high rates of revision required after expander-based reconstruction and the potential impact that this may have on future cost. In contrast to these findings, Lemaine *et al*.^17^ found that immediate unilateral implant-based reconstruction was associated with a higher 2-year cumulative cost of care compared with autologous reconstruction. All three studies defined the autologous group heterogeneously and included several types of procedure in their autologous reconstruction group. Furthermore, because only 2 to 3 years of follow-up were possible with these studies, it is difficult to draw conclusions about the true long-term cumulative cost of care beyond this time. Other North American studies have attempted to assess the cost-effectiveness of various breast reconstruction options, but have significant methodological limitations. Two studies^31,32^ did not include autologous reconstruction options, five studies^32–36^ used expert opinion to derive the health-related utilities, and some studies^34,36–40^ used a time horizon of only 1 year in their analysis. As such, the results from these cost-effectiveness studies are unlikely to be reliable. The current study used comprehensive real-world longitudinal data from a representative national cohort to provide much-needed information regarding the long-term healthcare costs of the five most commonly performed breast reconstruction procedures by considering costs up to 8 years after surgery. The quality of this routinely collected national data is subject to regular data-quality checks, and importantly it is used to reimburse hospital activity so data completeness on key variables is high. It is likely to provide an accurate representation of healthcare costs in an NHS setting.

There are several limitations to this study. A number of factors may have resulted in the true cost of inpatient care being underestimated. For example, treatments that are privately funded and carried out in private settings were not included in the analysis, but geographically diverse routine inpatient data from the NHS hospitals in England were included. Much adjuvant therapy is given on an outpatient basis, so these figures underestimate both the use and costs associated with additional cancer treatments, particularly radiotherapy. Similarly, other outpatient attendances were excluded due to incomplete data. This is a key limitation, as many procedures, including expansion of tissue expanders and management of short and long-term complications, are undertaken in the outpatient setting. Therefore, the true cost of the reconstruction may have been underestimated, particularly in the prosthetic reconstruction groups.

There is also uncertainty related to the complexity of coding. The coding of revisions and secondary reconstructions within the HES-APC data set was challenging and relied on clinical interpretation and multiple iterations of coding combinations to identify and classify procedures. Batches of data were sequentially checked, and definitions of codes refined as the coding classifications were developed, but it is possible that some procedures were missed or classified incorrectly. For example, specific events of interest, such as removal of an autologous flap due to flap failure, do not have an exact OPCS (Operating Procedure Codes Supplement) code. As flap failure would occur in the immediate perioperative interval, OPCS codes describing additional procedures performed during the index inpatient episode were explored to identify codes indicative of flap failure/removal. Any subsequent reconstruction occurring after a free-flap reconstruction was, however, considered a secondary reconstruction, which may have slightly overestimated rates of secondary reconstruction, and hence the costs in this group. Similarly, it was seen that codes relating to ‘protheses’ and ‘tissue expanders’ were sometimes used interchangeably. This may have been particularly relevant for patients who were having an adjustable implant, such as a Becker device, inserted. These devices are definitive prostheses with a silicone component and a saline chamber that can be expanded via a removable port. As such, they should have been included in the implant group, but it is possible they could have been miscoded as expanders and consequently included in the expander study group, potentially impacting the associated procedure costs. These patients would also have required a procedure to remove the port. There is no specific OPCS code for port removal in the context of a breast implant, but it is likely that this was captured within the comprehensive list of codes indicative of revisions that was developed for the study and costed appropriately.

This study specifically only considered the costs of IBR after unilateral mastectomy for breast cancer and women undergoing bilateral procedures were excluded. This was because the bilateral group was small, precluding meaningful comparative analysis. While the cost of the index bilateral reconstruction would be higher, women undergoing bilateral surgery may require fewer revisions and/or contralateral procedures over time to address asymmetry. The impact on the overall costs is unknown and further work is required to explore the costs of reconstruction in this specific patient group, especially as mainstream genetic testing is increasingly available.

The costs described in this study relate to care provided between 2009 and 2015, and practice has evolved significantly in recent years. Implant-based breast reconstructions are now the main type of reconstruction performed and numbers of LD flaps have declined. Perhaps, more importantly, patients undergoing free-flap reconstruction now benefit from enhanced recovery programmes that reduce the length of stay to as little as 3 days and consequently the associated costs of care^41^. New approaches to implant-based reconstruction also allow day-case reconstruction^42^, although the meshes used in these procedures are expensive and will add to the costs. These costs are not captured in our analyses, but would typically add between €1200 and €3500 to the cost of the index procedure, depending on the product used. This is likely to make the current costs of the index implant-based and AFF reconstructions more broadly comparable. Finally, the costs reported in this study reflect UK practice and may not be generalizable to other settings.

This study provides much-needed data regarding the long-term secondary healthcare costs of the most common approaches to IBR, but cost is only one of many considerations when evaluating different breast reconstruction procedures. Breast reconstruction is offered to improve the quality of life for women with breast cancer, and it is vital to integrate patient satisfaction with outcome and the impact of the surgery on key patient-reported outcomes to determine which reconstruction procedures represent ‘value for money’. This is important because different procedures vary in how they impact patient well-being, and this has been shown to change over time^43^. In particular, there is increasing evidence from both this^44^ and other studies^43^ that the long-term patient-reported outcomes of AFF reconstructions are superior to those after implant-based procedures. Therefore, although free-flap reconstruction may be the most expensive breast reconstruction option, it may be a more cost-effective reconstruction than implants, which were found to require more revisions and secondary reconstructions over time.

Health-related quality of life (utility) values are key parameters in the assessment of the cost-effectiveness of interventions. Although cost-effectiveness models offer a means of comparing the long-term efficacy of different interventions^45^, determining the cost-effectiveness of breast reconstruction is challenging, as generic measures (such as the EQ-5D-5L) used to calculate health-related utilities may not be sufficiently sensitive for use in this population^46^. Preference-sensitive measures may provide a solution and a health utility module for the breast reconstruction specific BREAST-Q is being developed, but is not yet ready for use^47^. Therefore, further research is needed to compare both the costs and outcomes of implant-based and autologous breast reconstruction procedures in the UK to inform decision-making regarding the most cost-effective option, and the cost evidence presented in this paper should prove useful for future cost-effectiveness analyses.

The present study demonstrates clear differences in the secondary-care costs of different approaches to IBR and how these vary over time. Expander-based procedures may represent the least expensive option, but the costs of revisions and secondary reconstructions are high and likely to increase further over time. By contrast, although abdominal flap reconstruction was seen to be the most expensive procedure overall, the current costs of care will be lower due to improvements in patient-care pathways and reduced rates of complications. Furthermore, the reduced need for revisions and secondary reconstructions after AFF, together with high levels of long-term patient satisfaction after autologous reconstruction, may mean that this option offers best use of NHS resources in the longer term.

## Collaborators

**Brighter Study Group** Chris Holcombe (Professor of Breast Surgery, Royal Liverpool University Hospital, Liverpool UK); Joe O’Donoghue, (Consultant Plastic Surgeon, Newcastle Upon Tyne Hospitals NHS Foundation Trust, Newcastle, UK); John Browne (Professor of Health Services Research, University College Cork, Ireland); Carmel Gulliver-Clarke (Consultant Nurse, Western Sussex Hospitals NHS Foundation Trust, Sussex, UK); Ranjeet Jeevan (Consultant Plastic Surgeon, Manchester University Hospitals NHS Foundation Trust, Manchester, UK); Paul White (Associate Professor of Applied Statistics, University of the West of England, Bristol, UK); Mairead Mackenzie (Trustee, Patient Involvement, Independent Cancer Patients Voice, UK); Patricia Fairbrother (Trustee, Independent Cancer Patients Voice, UK).

## Supplementary Material

znad149_Supplementary_Data

## Data Availability

The study is based on NHS Hospital Episode Statistics data and was provided within the terms of an NHS Digital data-sharing agreement. The data do not belong to the authors and may not be shared by the authors, except in aggregate form for publication. Data can be obtained by submitting a research request via the NHS Digital Data Access Request Service.
