# Supplementary material for: Secondary healthcare costs after mastectomy and immediate breast reconstruction for women with breast cancer in England: population-based cohort study
Source: Br J Surg. 2023 Jun 12;110(9):1171–9. doi: 10.1093/bjs/znad149 (PMC10416683; doi:10.1093/bjs/znad149)
Supplement: znad149_Supplementary_Data [file znad149_supplementary_data.docx]

**Title: Secondary healthcare costs following mastectomy and immediate breast reconstruction for women with breast cancer in England: population-based cohort study**

**Authors:** Syed Mohiuddin^1†^, William Hollingworth^2,3^, Joel Glynn^2^, Tim Jones^2,3^, Leigh Johnson^2^, Shelley Potter^4,5*^ On behalf of the Brighter Study Group^6^

^1^Centre for Guidelines, National Institute for Health and Care Excellence, London, UK

^2^Population Health Sciences, Bristol Medical School, University of Bristol, Bristol, UK

^3^NIHR ARC West, University Hospitals Bristol and Weston NHS Foundation Trust, Bristol, UK

^4^Translational Health Sciences, Bristol Medical School, University of Bristol, Bristol, UK

^5^Bristol Breast Care Centre, Southmead Hospital, Bristol, UK

^6^The members of BRIghterR study group investigators are listed in author contributions

^†^This work was carried out while working for Bristol Medical School, University of Bristol, Bristol, UK

**Corresponding author.** Shelley Potter **Email:** shelley.potter@bristol.ac.uk

Translational Health Sciences, Bristol Medical School, University of Bristol, Bristol, UK

**Twitter** @drshelleypotter

**Supplementary Materials - Index**

| **Supplementary Figures and Tables** |  |
| --- | --- |
| Table S1: Immediate breast reconstruction study group types | *pag. 2* |
| Table S2. Revision procedures – OPCS codes | *pag. 3* |
| Table S3. Secondary reconstruction procedures – OPCS codes | *pag. 4* |
| Table S4. Completion procedures – OPCS codes | *pag. 5* |

**Supplementary Figures and Tables**

**Table S1.** Immediate breast reconstruction study group types

| **Immediate BR type** | **OPCS codes for inclusion** | **3-year follow-up**  **n (%)** | **8-year follow-up**  **n (%)** |
| --- | --- | --- | --- |
| Implant | B30.1 without (S48.2, B29.1, B29.2, B29.3, B29.4, B29.6, B29.7, B38.1, B38.2, B38.8, B38.9, B39.1, B39.2, B39.3, B39.5, B39.8, AND B39.9) | 5,192  (30.7%) | 1074  (21.1%) |
| Expander | S48.2 without (B30.1, B29.1, B29.2, B29.3, B29.4, B29.6, B29.7, B38.1, B38.2, B38.8, B38.9, B39.1, B39.2, B39.3, B39.5, B39.8, AND B39.9) | 2,826  (16.7%) | 842  (16.5%) |
| LD flap- | B29.1 without (B30.1 AND S48.2) | 2,372  (14.0%) | 993  (19.5%) |
| LD flap+ | B29.1 with (B30.1 OR S48.2) | 3,109  (18.4%) | 1235  (24.2%) |
| AFF | B39.1 or B39.3 | 3,391  (20.1%) | 950  (18.6%) |
|  |  | **16,890** | **5,094** |

OPCS (Office of Population Censuses and Surveys Classification of Interventions and Procedures)

LD flap- (autologous pedicled latissimus dorsi flap without implant/expander)

LD flap+ (pedicled latissimus dorsi flap with implant and/or expander)

AFF (abdominal free flap)

**Table S2.** Revision procedures – OPCS codes

OPCS (Office of Population Censuses and Surveys Classification of Interventions and Procedures); LD flap- (autologous pedicled latissimus dorsi flap without implant/expander); LD flap+ (pedicled latissimus dorsi flap with implant and/or expander); AFF (abdominal free flap); NEC (not elsewhere classified); NOC (not otherwise classifiable)

**Table S3.** Secondary reconstruction procedures – OPCS codes

OPCS (Office of Population Censuses and Surveys Classification of Interventions and Procedures)

LD flap- (autologous pedicled latissimus dorsi flap without implant/expander)

LD flap+ (pedicled latissimus dorsi flap with implant and/or expander)

AFF (abdominal free flap)

NEC (not elsewhere classified)

**Table S4.** Completion procedures – OPCS codes

OPCS (Office of Population Censuses and Surveys Classification of Interventions and Procedures)

LD flap- (autologous pedicled latissimus dorsi flap without implant/expander)

LD flap+ (pedicled latissimus dorsi flap with implant and/or expander)

AFF (abdominal free flap)

NEC (not elsewhere classified)
